# Parental involvement in infection prevention and control in low- and middle-income country neonatal units: a scoping review protocol

**DOI:** 10.1136/bmjopen-2024-093967

**Published:** 2025-04-17

**Authors:** Lydia Davidson, Chikomborero Kitikiti, Hannah Blencowe, Felicity Fitzgerald, Sarah Moxon, Gwendoline Chimhini, Rudo Chingono

**Affiliations:** 1 MARCH Centre, London School of Hygiene & Tropical Medicine, London, UK; 2 The Health Research Institute, Biomedical Research and Training Institute, Harare, Zimbabwe; 3 Division of Paediatrics, Harare Central Hospital, Harare, Zimbabwe; 4 Department of Infectious Diseases, Imperial College London, London, UK; 5 Department of Child Adolescent and Women’s Health, University of Zimbabwe Faculty of Medicine and Health Sciences, Harare, Zimbabwe

**Keywords:** Infection control, NEONATOLOGY, Caregivers

## Abstract

**Abstract:**

**Introduction:**

Neonatal sepsis is a key contributor to neonatal mortality worldwide, and low- and middle-income countries (LMIC) are disproportionately affected. With antimicrobial resistance challenging effective treatment of neonatal sepsis, it is increasingly urgent to improve infection prevention and control (IPC) in LMIC neonatal units (NNU) and reduce transmission of infections. One pathway to improvement which merits further exploration is the collaboration with families to build an IPC intervention.

Families are constantly present on neonatal units, and much of the hands-on care for their newborns is given by them. For IPC to be effective, families must adhere to IPC standards within the NNU, but furthermore, any IPC intervention implemented must be feasible and acceptable for families as well as the hospital staff as this will increase uptake and effectiveness of the intervention. This scoping review aims to provide an overview of parental involvement in infection prevention and control in low- and middle-income setting neonatal units.

**Methods and analysis:**

This protocol was developed in line with the Joanna Briggs Institute recommendations. Searches will be carried out on six databases (Medline, CINAHL, Global Health, EMBASE, Web of Science and Global Index Medicus), and reference searching will be carried out on included studies. The search will be carried out from 2000 to present (end date 28/02/2024), and included languages will be English, French, Spanish and Portuguese. Screening and data extraction will be performed independently by two reviewers, with a third reviewer to resolve conflicts. Results will be reported by narrative synthesis of each sub-question in line with the Preferred Reporting Items for Systematic Reviews and Meta-Analyses extension for Scoping Reviews guidelines.

**Ethics and dissemination:**

This study will be carried out using already published data exclusively and therefore does not require further ethical approval. Results will be disseminated through peer-reviewed publications and conference presentations and through engagement with peers and relevant stakeholders.

**Trial registration number:**

Registered with Open Science Framework - https://osf.io/snc7a/?view_only=8ffc39d837594b4388c7394a838c3a9e

**STRENGTHS AND LIMITATIONS OF THIS STUDY**

- Despite the constant presence of parents within neonatal units, there is limited information on parental involvement in infection prevention and control (IPC) in low- and middle-income country neonatal units. This scoping review provides an overview of existing evidence and consideration of further areas of exploration.
- The search strategy is comprehensive including broad search terms to capture as much information as possible and covers six databases, four languages and hand-searching references.
- A scoping review is more likely to identify the breadth of evidence around parental involvement in IPC, as both interventions and study methodologies are likely to be heterogeneous and the strict criteria necessary for a systematic review may limit which studies are identified.
- There may be publications in languages other than those searched here.
- This study focuses on the neonatal unit only, due to the unique nature of parental involvement in this setting but acknowledges that infections can be contracted within the labour ward, at the time of delivery or in the community.

## Introduction

 Neonatal sepsis is a key contributor to neonatal mortality worldwide, and low- and middle-income countries (LMIC) are disproportionately affected.^1^ Although neonatal sepsis is in principle a treatable illness, and the treatment is well known, there are multiple barriers to preventing, identifying and managing sepsis in LMIC. Due to these challenges, neonatal sepsis in LMIC frequently results in significant mortality, morbidity and healthcare costs.^2^

Within healthcare settings in LMIC, it is challenging to identify sepsis as clinical signs are often non-specific and subtle, reliant on close observation to identify.^3^ Additionally, there are often high patient-to-staff ratios, meaning that close observation is extremely challenging.^4^ Blood cultures are recognised as the current gold standard required to confirm the diagnosis of sepsis and identify the causative pathogen—in LMIC, blood culture access is limited and inconsistent. This is in part due to a lack of resources but also due to challenges with the supply chain of consumables, availability of staff with the specialist skills necessary and relative cost of processing a blood culture within the healthcare system.^5 6^ Where blood cultures are available, there is often a significant delay in results or high rates of contamination, both impacting the clinical use and challenging interpretation.

Although neonatal infections are in principle treatable, increasing antimicrobial resistance is making treatment of sepsis and other bacterial infections increasingly difficult in LMIC. Indeed, the WHO recommended first-line treatment (a combination of Ampicillin and Gentamicin) is not effective in an increasing number of cases.^7^ A recent global review identified that 62.9% of positive blood cultures were Gram-negative organisms commonly resistant to WHO-recommended regimens.^8^ A meta-analysis reviewing the aetiology of invasive bacterial infections in neonates in sub-Saharan Africa indicated that in this region, Gram negative bacteria are the predominant cause of early onset sepsis, and combined with increasing antimicrobial resistance, this leads to a limited effectiveness of WHO regimens.^7^ This has, in part, been driven by antimicrobial use. For example, in Malawi, rates of resistance of Gram-negative pathogens to all first-line antimicrobials have increased dramatically since the introduction of ceftriaxone in the early 2000s.^9^

The pathogen *Klebsiella pneumoniae* is increasingly being recognised as a significant threat to global health, and in some settings carries a 70% case fatality rate.^10^ Reported resistance levels vary across regions and settings. Other pathogens are predominant in different areas, making the first-line choice of antibiotic challenging for clinicians, as the recommended first-line antibiotics are not effective against the predominant pathogens in the region.^7^ *Klebsiella* has traditionally been associated with late-onset sepsis (sepsis >72 hours after delivery) and is associated with healthcare-associated infections. However, it is now increasingly being identified in the first 24 hours after birth, indicating a change in the epidemiology of the timing of the onset of infection with healthcare-associated infections being transmitted in the immediate hours after birth in labour and postnatal wards.^11^ Indeed, Zaidi *et al* have recommended that neonatal sepsis following any facility-based delivery be classified as a healthcare-associated infection, regardless of the time of onset, with this being recognised in Strengthening the Reporting of Observational Studies in Epidemiology for Newborn Infection guidelines by moving away from a binary classification of early versus late to documenting the time of onset of any infection postdelivery.^12 13^

It is increasingly urgent to improve infection prevention and control (IPC) in LMIC neonatal units (NNU) and reduce transmission of infections. One pathway to improvement that has yet to be underexplored is the collaboration with families to build an IPC intervention. Families are constantly present on neonatal units, and much of the hands-on care for their newborns is given by them. This provision of care, however, is informal with limited education for families in how to safely provide care for their newborns.^4 14^ Care practices that require hygiene measures (eg, handwashing, feeding, changing diapers) are often informally adopted through peer learning among mothers of newborns.

A scoping review on bundles of care addressing healthcare-associated infections (HCAI) in LMIC NNUs both highlights some strategies that may be used to involve families in IPC and the very limited involvement of parents thus far. Identified IPC interventions were analysed by each part of an intervention, called an ‘element’. In total, four separate elements were found from 44 papers that related to parental involvement. Two elements related to feeding, one to skin-to-skin contact and one to empowering mothers to engage in routine care.^15^

For IPC to be effective, families must adhere to IPC standards within the NNU, but furthermore, any IPC intervention implemented must be feasible and acceptable for families as well as the hospital staff as this will increase uptake of and adherence to the intervention.

### Research questions

At present, there is scarce literature on how parents have been involved in IPC in LMIC NNUs. This review aims to review the scope of the evidence around this topic by addressing three sub-questions.

1. How have parents been involved in designing interventions in LMIC NNUs?
2. How have parents been involved in the delivery of IPC interventions?
3. What is the parental experience of hygiene and care in the NNU in LMIC?

### Rationale for a scoping review

A scoping review was chosen because at present there is limited information on parental involvement in IPC on LMIC NNUs, insufficient information to form a specific and answerable question for a systematic review, and identified methods and interventions are likely to be heterogeneous and unsuited for a systematic review. There are systematic reviews of connected concepts, for example, effective interventions to prevent infections, gaps in IPC in LMIC NNUs, parental roles in hand hygiene in paediatrics or antimicrobial resistance, but none directly about parents and IPC in LMIC.^7 11 16 17^

### Objectives

To evaluate published literature on parental involvement in the designing and implementation of IPC interventions in LMIC NNUs.

To evaluate published literature on parental experience of IPC interventions (design, delivery and impact) in LMIC NNUs.

## Methods

This review protocol has been written in compliance with the Joanna Briggs Institute manual and will be registered with the Open Science Framework database.^18 19^ The proposed review will follow the Preferred Reporting Items for Systematic Reviews and Meta-Analyses extension for Scoping Reviews (PRISMA-ScR) Checklist.^20^ Research objectives, inclusion and exclusion criteria and search methods are described and defined prior to study commencement. The end date for searches is 28/02/2024.

### Participants

The target population of this review are guardians (parents, families or non-medical caregivers) of both term and preterm neonates in hospitals in low- and middle-income countries.

### Concept

The key concept of this review is infection prevention and control interventions in LMIC neonatal units.

### Context

The context of this review is inpatient neonatal units in LMIC.

## Inclusion and exclusion criteria

**Table:** 

Criteria	Inclusion	Exclusion
Care context	Studies carried out in the neonatal unit.	Studies carried out in the community and in paediatric wards.
Publication date	Studies published from 2000 to current.	Studies published prior to 2000.
Language	English, French, Portuguese, Spanish.	Literature not available in English, French, Portuguese or Spanish.
Participants	Studies that report on parents’ involvement in the design, implementation or experience of IPC interventions in NNU.	Studies with nil evidence or documentation of parental participation in design, implementation or experience of IPC interventions in NNU.
Setting	Studies must be carried out in LMIC.	Studies not carried out in LMIC.

### Rationale

*Study type:* The concept of study type will include qualitative studies to identify studies that report on family experience of IPC, and cohort studies and programme evaluation will capture intervention development and implementation related to parents in the NNU.

*Care context:* Paediatric wards frequently have a different layout, staffing pattern and patient type compared with neonatal wards—this will not provide the information needed to improve IPC in NNUs. Studies aimed at care in the community will not provide information on parental involvement with IPC in LMIC NNUs.

*Publication date:* This time frame will allow for gathering as much evidence as possible while ensuring that it remains relevant. Key understanding and evidence on neonatal infection have been gathered since 2000.

*Language:* The majority of studies are published in English, including French; this will ensure that French-speaking African countries are included, and the inclusion of Portuguese and Spanish will ensure that LMIC in Latin America are included.

*Setting:* The context of neonatal care is very different in high-income country settings. Availability of resources, such as power, water and antibiotics, makes the kinds of IPC interventions likely to be different. Although IPC principles remain the same, the challenges faced and therefore interventions needed are not likely to be transferable across contexts. Therefore, this study will include only studies carried out in LMIC. The Medline filter for low- and middle-income countries will be applied and adapted to other databases. The filter has been created using countries in the World Bank lending groups of low- and middle-income countries for 2021.

### Search strategy

The search strategy including the concepts of parents, IPC and neonatal unit was created to capture all three study sub-questions.

A test search has been created in Ovid Medline and reviewed by a librarian (online supplemental file 1). The keywords and synonyms are below in table 1.

**Table 1:** Keywords and synonyms

Parents	IPC	Neonatal unit
Exp Parents/Exp Family/MotherFatherCarerCaregiverGuardianParentFamilyFamilies	exp Infection Control/exp Hygiene/exp Cross Infection/*Neonatal Sepsis/WASHHAIIPCHygieneNeonatal infectionNeonatal sepsisInfection adj2 (prevention or control)	exp Infant, Newborn/exp Intensive Care Units, Neonatal/NBU NNU NICU or SCBU or NNU orSpecial care baby unitNewborn*Neonat*

"*"indicates truncation of a search term.

Titles and abstracts will be screened for inclusion criteria concepts and keywords. Subsequent full-text screening will then be carried out based on inclusion criteria.

#### Filters applied

- Medline all countries LMIC
- Study type—qualitative to capture experience, interventional to capture IPC interventions from https://libguides.sph.uth.tmc.edu/search_filters/ovid_medline_filters

#### Limits applied

- 2000 to present
- English, French, Portuguese and Spanish

### Databases

- Medline
- CINAHL
- Global Health
- EMBASE
- Web of Science
- Global Index Medicus

The search strategy was created in Medline and will be applied to EMBASE and Global Health and adapted for CINAHL, Web of Science and Global Index Medicus. This study will be carried out by two independent reviewers, with a third to discuss disagreements. All results will be imported to EndNote 21 and duplicates removed. Titles and abstracts will then be searched against the selection criteria on Rayyan. A full text review for inclusion will then be carried out. Reference searching of identified articles will be carried out. Grey literature from reference searching will be included if inclusion criteria are met; however, no formal grey literature search will be carried out.

### Charting

General data points to be extracted

- Author(s)
- Year of publication
- Country where the study was conducted
- Study type
- Outcome measures

Data for studies relevant to Q1

- Stage of research parents were involved in
- What form did parental involvement take, for example, survey, focus group discussions, in-depth interviews or workshops

Data for studies relevant to Q2

- The parental role in delivering the intervention, for example, carrying out chlorhexidine washes, skin care, environmental cleaning.

Data for studies relevant to Q3

- Themes identified in parental experience related to IPC

Qualitative content analysis will be descriptive and allow an understanding of the reported experience of parents in LMIC NNU and identify common themes across LMIC, as well as the range of experience reported.

### Strengths and limitations

This study will provide an overview of the involvement of parents in low- and middle-income country neonatal units. At present, although parents are frequently providing hands-on care to newborns in these settings, there is little evidence on how they are involved with infection prevention and control. This protocol will enable a broad range of evidence to be collected, as the search terms are broad, six databases are included and four languages are used. Additionally, a scoping review allows the inclusion of heterogeneous studies so the breadth of the evidence can be understood.

Limitations of this study are that it does not include a wider range of languages—there may be papers in languages other than English, French, Spanish and Portuguese. Additionally, this scoping review focuses solely on the neonatal unit. The aim of this is to develop a review that will underpin research and policy on IPC within the NNU. However, there may be evidence on parental involvement in hygiene and infection control at home or in other settings that may be related and informative.

### Dissemination

The findings of this review will be disseminated to key stakeholders through publication and presentations. The findings will also be used to inform future policy and research aimed at improving infection prevention and control in low- and middle-income neonatal units.

### Patient and public involvement

We pla to utilise the results of this study to underpin future collaborative research with families in neonatal units, as well as to engage patient representatives for communication or dissemination of results.

## Supplementary material

10.1136/bmjopen-2024-093967 online supplemental file 1
