# Supplementary material for: Parental involvement in infection prevention and control in low- and middle-income country neonatal units: a scoping review protocol
Source: BMJ Open. 2025 Apr 17;15(4):e093967. doi: 10.1136/bmjopen-2024-093967 (PMC12007048; doi:10.1136/bmjopen-2024-093967)
Supplement: online supplemental file 1 [file bmjopen-15-4-s001.docx]

Ovid MEDLINE(R) ALL <1946 to February 27, 2024>

1 Developing Countries/ 81840

2 ((developing or less* developed or under developed or underdeveloped or middle income or low* income) adj (economy or economies)).ti,ab. 1073

3 ((developing or less* developed or under developed or underdeveloped or middle income or low* income or underserved or under served or deprived or poor*) adj (countr* or nation? or population? or world)).ti,ab. 141471

4 (low* adj (gdp or gnp or gross domestic or gross national)).ti,ab. 376

5 (low adj3 middle adj3 countr*).ti,ab. 33372

6 (lmic or lmics or third world or lami countr*).ti,ab. 14087

7 transitional countr*.ti,ab. 183

8 global south.ti,ab. 1245

9 "africa south of the sahara"/ 13586

10 africa, central/ 1401

11 africa, eastern/ 4451

12 africa, southern/ 2705

13 africa, western/ 6418

14 ("Africa South of the Sahara" or sub-Saharan Africa or subSaharan Africa).ti,ab. 30516

15 Central Africa.ti,ab. 3913

16 Eastern Africa.ti,ab. 1332

17 Southern Africa.ti,ab. 5469

18 Western Africa.ti,ab. 1078

19 "Democratic People's Republic of Korea"/ 308

20 (North Korea or (Democratic People* Republic adj2 Korea)).ti,ab. 559

21 Cambodia/ 3918

22 Cambodia.ti,ab. 4942

23 Indonesia/ 14095

24 (Indonesia or Dutch East Indies).ti,ab. 19507

25 (Kiribati or Gilbert Islands or Phoenix Islands or Line Islands).ti,ab. 310

26 Laos/ 2294

27 (Laos or (Lao adj1 Democratic Republic)).ti,ab. 2610

28 Micronesia/ 1283

29 Micronesia.ti,ab. 768

30 Mongolia/ 2134

31 Mongolia.ti,ab. 5944

32 Myanmar/ 3272

33 (Myanmar or Burma).ti,ab. 5785

34 Papua New Guinea/ 3776

35 (Papua New Guinea or German New Guinea or British New Guinea or Territory of Papua).ti,ab. 5049

36 Philippines/ 9748

37 (Philippines or Philippine Islands).ti,ab. 11121

38 "Independent State of Samoa"/ 251

39 ((Samoa not American Samoa) or Western Samoa or Navigator Islands or Samoan Islands).ti,ab. 719

40 Solomon Islands.ti,ab. 984

41 Timor-Leste/ 279

42 (Timor-Leste or East Timor or Portuguese Timor).ti,ab. 687

43 Vanuatu/ 430

44 (Vanuatu or New Hebrides).ti,ab. 858

45 Vietnam/ 15164

46 (Viet Nam or Vietnam or French Indochina).ti,ab. 20791

47 American Samoa/ 215

48 American Samoa.ti,ab. 427

49 exp China/ 283148

50 China.ti,ab. 310459

51 Fiji/ 1147

52 Fiji.ti,ab. 2396

53 Malaysia/ 18370

54 (Malaysia or Malayan Union or Malaya).ti,ab. 22752

55 Marshall Islands.ti,ab. 362

56 Nauru.ti,ab. 172

57 Thailand/ 31377

58 (Thailand or Siam).ti,ab. 34812

59 Tonga/ 294

60 Tonga.ti,ab. 577

61 (Tuvalu or Ellice Islands).ti,ab. 92

62 Kyrgyzstan/ 1411

63 (Kyrgyzstan or Kyrgyz Republic or Kirghizia or Kirghiz).ti,ab. 1316

64 Tajikistan/ 811

65 Tajikistan.ti,ab. 784

66 Ukraine/ 17387

67 Ukraine.ti,ab. 7234

68 Uzbekistan/ 2013

69 Uzbekistan.ti,ab. 1421

70 Albania/ 970

71 Albania.ti,ab. 1364

72 Armenia/ 1604

73 Armenia.ti,ab. 1462

74 Azerbaijan/ 1304

75 Azerbaijan.ti,ab. 1758

76 "Republic of Belarus"/ 2158

77 (Belarus or Byelarus or Byelorussia or Belorussia).ti,ab. 1815

78 Bosnia-Herzegovina/ 2369

79 (Bosnia or Herzegovina).ti,ab. 2857

80 Bulgaria/ 6738

81 Bulgaria.ti,ab. 5075

82 "Georgia (Republic)"/ 2031

83 Georgia.ti,ab. not Georgia/ 7838

84 Kazakhstan/ 3175

85 (Kazakhstan or Kazakh).ti,ab. 4098

86 Kosovo/ 314

87 Kosovo.ti,ab. 1139

88 Moldova/ 760

89 Moldova.ti,ab. 725

90 Montenegro/ 297

91 Montenegro.ti,ab. 1078

92 "Republic of North Macedonia"/ 656

93 North Macedonia.ti,ab. 269

94 Romania/ 11073

95 Romania.ti,ab. 8074

96 exp Russia/ 56629

97 "Russia (Pre-1917)"/ 5987

98 USSR/ 42860

99 (Russia or Russian Federation or USSR or Union of Soviet Socialist Republics or Soviet Union).ti,ab. 34103

100 Serbia/ 3849

101 Serbia.ti,ab. 5805

102 Turkey/ 40946

103 (Turkey.ti,ab. not animal/) or (Anatolia or Asia Minor).ti,ab. 33991

104 Turkmenistan/ 591

105 Turkmenistan.ti,ab. 445

106 Belize/ 649

107 (Belize or British Honduras).ti,ab. 1024

108 Bolivia/ 2868

109 Bolivia.ti,ab. 3912

110 El Salvador/ 968

111 El Salvador.ti,ab. 1539

112 Haiti/ 3634

113 (Haiti or Hayti).ti,ab. 3607

114 Honduras/ 1264

115 Honduras.ti,ab. 2148

116 Nicaragua/ 1638

117 Nicaragua.ti,ab. 2197

118 Argentina/ 18204

119 (Argentina or Argentine Republic).ti,ab. 21022

120 Brazil/ 119673

121 Brazil.ti,ab. 109718

122 Colombia/ 13648

123 Colombia.ti,ab. 17416

124 Costa Rica/ 4076

125 Costa Rica.ti,ab. 5864

126 Cuba/ 5387

127 Cuba.ti,ab. 5156

128 Dominica/ 116

129 Dominica.ti,ab. 592

130 Dominican Republic/ 1787

131 Dominican Republic.ti,ab. 2369

132 Ecuador/ 4789

133 Ecuador.ti,ab. 6477

134 Grenada/ 173

135 Grenada.ti,ab. 388

136 Guatemala/ 3371

137 Guatemala.ti,ab. 4336

138 Guyana/ 760

139 (Guyana or British Guiana).ti,ab. 1288

140 Jamaica/ 3645

141 Jamaica.ti,ab. 3592

142 Mexico/ 44720

143 (Mexico or United Mexican States).ti,ab. 53072

144 Panama/ 2597

145 Panama.ti,ab. 4818

146 Paraguay/ 942

147 Paraguay.mp. 2186

148 Peru/ 11101

149 Peru.ti,ab. 14199

150 Saint Lucia/ 77

151 (St Lucia or Saint Lucia or Iyonala or Hewanorra).ti,ab. 388

152 "Saint Vincent and the Grenadines"/ 59

153 (Saint Vincent or St Vincent or Grenadines).ti,ab. 679

154 Suriname/ 1020

155 (Suriname or Dutch Guiana).ti,ab. 771

156 Venezuela/ 5171

157 Venezuela.ti,ab. 5921

158 Algeria/ 3609

159 Algeria.ti,ab. 4300

160 Djibouti/ 256

161 (Djibouti or French Somaliland).ti,ab. 472

162 Egypt/ 17920

163 Egypt.ti,ab. 18726

164 Iran/ 38901

165 (Iran or Persia).ti,ab. 54241

166 Morocco/ 6692

167 Morocco.ti,ab. 7549

168 Tunisia/ 9346

169 Tunisia.mp. 12371

170 (Gaza or West Bank or Palestine).ti,ab. 3571

171 Iraq/ 5706

172 (Iraq or Mesopotamia).ti,ab. 9193

173 Jordan/ 5557

174 Jordan.ti,ab. 8868

175 Lebanon/ 5310

176 (Lebanon or Lebanese Republic).ti,ab. 6356

177 Libya/ 1242

178 Libya.ti,ab. 1569

179 Afghanistan/ 3864

180 Afghanistan.ti,ab. 7280

181 Bangladesh/ 14913

182 Bangladesh.ti,ab. 20276

183 Bhutan/ 702

184 Bhutan.ti,ab. 1153

185 exp India/ 120889

186 India.ti,ab. 136285

187 Nepal/ 10998

188 Nepal.ti,ab. 13940

189 Pakistan/ 23362

190 Pakistan.ti,ab. 28317

191 Sri Lanka/ 7283

192 (Sri Lanka or Ceylon).ti,ab. 9054

193 Maldives.ti,ab. [UPPER MIDDLE INCOME COUNTRIES IN SOUTH ASIA] 520

194 Angola/ 1164

195 Angola.ti,ab. 1801

196 Benin/ 1978

197 Benin.ti,ab. 4225

198 Cameroon/ 6689

199 (Cameroon or Kamerun or Cameroun).ti,ab. 8927

200 Cape Verde/ 263

201 (Cape Verde or Cabo Verde).ti,ab. 798

202 Comoros/ 385

203 (Comoros or Glorioso Islands or Mayotte).ti,ab. 752

204 Congo/ 2036

205 (Congo not ((Democratic Republic adj3 Congo) or congo red or crimean-congo)).ti,ab. 3059

206 Cote d'Ivoire/ 3628

207 (Cote d'Ivoire or Cote dIvoire or Ivory Coast).ti,ab. 4622

208 Eswatini/ 791

209 (eSwatini or Swaziland).ti,ab. 1218

210 Ghana/ 11673

211 (Ghana or Gold Coast).ti,ab. 16032

212 Kenya/ 20070

213 (Kenya or East Africa Protectorate).ti,ab. 23505

214 Lesotho/ 550

215 (Lesotho or Basutoland).ti,ab. 996

216 Mauritania/ 511

217 Mauritania.ti,ab. 772

218 Nigeria/ 34977

219 Nigeria.ti,ab. 37654

220 (Sao Tome adj2 Principe).ti,ab. 208

221 Senegal/ 6302

222 Senegal.ti,ab. 6721

223 Tanzania/ 14281

224 (Tanzania or Tanganyika or Zanzibar).ti,ab. 17934

225 Zambia/ 5660

226 (Zambia or Northern Rhodesia).ti,ab. 6972

227 Zimbabwe/ 6679

228 (Zimbabwe or Southern Rhodesia).ti,ab. 7104

229 Botswana/ 2245

230 (Botswana or Bechuanaland or Kalahari).ti,ab. 3347

231 Equatorial Guinea/ 325

232 (Equatorial Guinea or Spanish Guinea).ti,ab. 529

233 Gabon/ 1654

234 (Gabon or Gabonese Republic).ti,ab. 2081

235 Mauritius/ 633

236 (Mauritius or Agalega Islands).ti,ab. 1196

237 Namibia/ 1340

238 (Namibia or German South West Africa).ti,ab. 2107

239 South Africa/ 50441

240 (South Africa or Cape Colony or British Bechuanaland or Boer Republics or Zululand or Transvaal or Natalia Republic or Orange Free State).ti,ab. 45773

241 Syria/ 2678

242 (Syria or Syrian Arab Republic).ti,ab. 2989

243 Yemen/ 1603

244 Yemen.ti,ab. 2350

245 Burkina Faso/ 4097

246 (Burkina Faso or Burkina Fasso or Upper Volta).ti,ab. 5517

247 Burundi/ 732

248 (Burundi or Ruanda-Urundi).ti,ab. 1164

249 Central African Republic/ 853

250 (Central African Republic or Ubangi-Shari).ti,ab. 1204

251 Chad/ 839

252 Chad.ti,ab. 1554

253 "Democratic Republic of the Congo"/ 5166

254 (((Democratic Republic or DR) adj2 Congo) or Congo-Kinshasa or Belgian Congo or Zaire or Congo Free State).ti,ab. 5617

255 Eritrea/ 428

256 Eritrea.ti,ab. 729

257 Ethiopia/ 20320

258 (Ethiopia or Abyssinia).ti,ab. 29158

259 Gambia/ 2738

260 Gambia.ti,ab. 2803

261 Guinea/ 1350

262 (Guinea not (New Guinea or Guinea Pig* or Guinea Fowl or Guinea-Bissau or Portuguese Guinea or Equatorial Guinea)).ti,ab. 3372

263 Guinea-Bissau/ 1044

264 (Guinea-Bissau or Portuguese Guinea).ti,ab. 1200

265 Liberia/ 1425

266 Liberia.ti,ab. 1973

267 Madagascar/ 3973

268 (Madagascar or Malagasy Republic).ti,ab. 5826

269 Malawi/ 6915

270 (Malawi or Nyasaland).ti,ab. 9336

271 Mali/ 2759

272 Mali.ti,ab. 4509

273 Mozambique/ 3128

274 (Mozambique or Mocambique or Portuguese East Africa).ti,ab. 4788

275 Niger/ 1462

276 (Niger not (Aspergillus or Peptococcus or Schizothorax or Cruciferae or Gobius or Lasius or Agelastes or Melanosuchus or radish or Parastromateus or Orius or Apergillus or Parastromateus or Stomoxys)).ti,ab. 4231

277 Rwanda/ 3243

278 (Rwanda or Ruanda).ti,ab. 4353

279 Sierra Leone/ 1995

280 (Sierra Leone or Salone).ti,ab. 3003

281 Somalia/ 1939

282 (Somalia or Somaliland).ti,ab. 2084

283 South Sudan/ 292

284 South Sudan.ti,ab. 836

285 Sudan/ 5250

286 Sudan.ti,ab. 9817

287 Togo/ 1302

288 (Togo or Togolese Republic or Togoland).ti,ab. 1858

289 Uganda/ 15935

290 Uganda.ti,ab. 19793

291 or/1-290 [ALL LMICs] 1966695

292 (mother* or father* or caregiver* or guardian* or carer* or parent* or famil*).mp. 2264195

293 exp Parents/ 147011

294 Family/ 85591

295 292 or 293 or 294 2264195

296 exp Infection Control/ 71405

297 exp Hygiene/ 46129

298 exp cross infection/ 65560

299 *Neonatal Sepsis/ 1325

300 (hygiene or neonatal infection* or neonatal sepsis* or WASH or IPC or HAI).mp. [mp=title, book title, abstract, original title, name of substance word, subject heading word, floating sub-heading word, keyword heading word, organism supplementary concept word, protocol supplementary concept word, rare disease supplementary concept word, unique identifier, synonyms, population supplementary concept word, anatomy supplementary concept word] 133176

301 (Infection adj2 (prevention or control)).mp. 66909

302 296 or 297 or 298 or 299 or 300 or 301 283660

303 exp Infant, Newborn/ 683615

304 exp Intensive Care Units, Neonatal/ 18603

305 (NICU or SCBU or NNU or NBU or special care baby unit or newborn* or neonat*).mp. 952132

306 303 or 304 or 305 954963

307 cohort studies/ or longitudinal studies/ or follow-up studies/ or prospective studies/ or retrospective studies/ or cohort.ti,ab. or longitudinal.ti,ab. or prospective.ti,ab. or retrospective.ti,ab. 3455037

308 Cross-Sectional Studies/ or Prevalence/ or (cross-sectional or prevalence or transversal).ti,ab,kw. 1451964

309 evaluation studies/ or evaluation studies as topic/ or program evaluation/ or validation studies as topic/ or ((pre- adj5 post-) or (pretest adj5 posttest) or (program* adj6 evaluat*)).ti,ab. or (effectiveness or intervention).ti,ab. 1887985

310 ((("semi-structured" or semistructured or unstructured or informal or "in-depth" or indepth or "face-to-face" or structured or guide) adj3 (interview* or discussion* or questionnaire*)) or (focus group* or qualitative or ethnograph* or fieldwork or "field work" or "key informant")).ti,ab. or interviews as topic/ or focus groups/ or narration/ or qualitative research/ 547721

311 Case-Control Studies/ or Control Groups/ or Matched-Pair Analysis/ or ((case* adj5 control*) or (case adj3 comparison*) or control group*).ti,ab. 978517

312 feasibility studies/ 85172

313 pilot projects/ 151254

314 exp program evaluation/ 85636

315 (program evaluat* or pilot intervention* or intervention* stud* or observation* stud* or ethnograph*).mp. 393258

316 307 or 308 or 309 or 310 or 311 or 312 or 313 or 314 or 315 7045942

317 291 and 295 and 302 and 306 and 316 897

318 limit 317 to (yr="2000 -Current" and (english or french or portuguese or spanish)) 764
