# Supplementary material for: Parental involvement in infection prevention and control in low- and middle-income country neonatal units: a scoping review protocol
Source: BMJ Open. 2025 Apr 17;15(4):e093967. doi: 10.1136/bmjopen-2024-093967 (PMC12007048; doi:10.1136/bmjopen-2024-093967)
Supplement: Supplementary file 2 [file bmjopen-2024-093967.reviewer_comments.pdf]

PEER REVIEW HISTORY

BMJ Open publishes all reviews undertaken for accepted manuscripts. Reviewers are asked to complete a checklist review form and are provided with free text boxes to elaborate on their assessment. These free text comments are reproduced below.

ARTICLE DETAILS

Title (Provisional)

Parental involvement in infection prevention and control in low- and middle-income country neonatal units: a scoping review protocol

Authors

Davidson, Lydia; Kitikiti, Chikomborero; Blencowe, Hannah; Fitzgerald, Felicity; Moxon, Sarah; Chimhini, Gwendoline; Chingono, Rudo

---

VERSION 1 - REVIEW

**Reviewer**	**1**
**Name**	**McGrath, Caitlin**
**Affiliation**	**Seattle Children's Hospital**
**Date**	**02-Jan-2025**
**COI**	**Non-financial associations:**

Committee work for the Society of Healthcare Epidemiology of America (SHEA); prior speaker at Society of Healthcare Epidemiology of America (SHEA) Spring Meeting and Quality Improvement Workshop and ID Week Conference.

This scoping review protocol by the author team presents an organized and thoughtful approach for addressing important questions about the roles of parents and families in infection prevention and control (IPC) in low- and middle- income (LMIC) countries. I commend the authors for devoting time and effort to research this important topic.

As detailed by the authors, little is known about parental and family involvement in IPC, and more knowledge about this topic is likely advance effective IPC strategies and interventions for newborns in LMIC. The presented protocol adheres to PRISMA-ScR guidelines. The research questions and inclusion and exclusion criteria are appropriate. Strengths include the inclusion of multiple languages of articles and article screening by multiple reviewers.

I recommend this protocol for publication with minor edits as detailed below.

Introduction:

• First paragraph: For the sentence with “neonatal sepsis is a treatable illness”, authors could also consider adding that while treatable, it can result in significant morbidity, mortality, and healthcare costs.

• Fourth paragraph: I would consider rewording the sentence starting with “Klebsiella” and ending with “healthcare-associated infections” to add some clarity or further detail. For example, rather than saying the “increasing problem of HAI” one could perhaps comment on the changing epidemiology of infection timing, etc.

• NNU: Would define this at time of first use in both the abstract and introduction.

Charting section:

• Would spelling out/defining the acronyms used in this section.

**Reviewer**	**2**
**Name**	**Piris-Borregas , Salvador**
**Affiliation**	**12 de Octubre University Hospital**
**Date**	**18-Feb-2025**
**COI**	**None**

Title: Parental involvement in infection prevention and control in

low- and middle-income country neonatal units: a scoping

review protocol

Manuscript ID bmjopen-2024-093967

This is a review to include families in teams that adhere to a specific protocol aimed at reducing the infection rate in lower-middle income neonatal units. The topic is interesting and innovative. The incorporation of families in clinical decision making is a worldwide trend with multiple benefits already known.

Page 3. General comment about the introduction. At no point in the introduction do the authors show possible strategies to reduce the nosocomial infection rate including the involvement of families. What strategies are they referring to? How can families be trained? Is the way families are trained systematised for all families equally or are individualised strategies tailored to the family situation being sought?

Page 3. Line 27. Please, clarify the reason why the Access to the blood culture is inconsistent. It is only a matter of resources? Could be also a matter of infection protocols routines implemented in the neonatal units?.

Page 3. Line 39. Please make reference in the introduction to the causes that may explain why 69% of cultures isolate gram-negative microorganisms that are not treated with the antibiotics recommended by the WHO.

Page 4. Line 43.Participants. Do the authors think that the measures to train families should be the same when the newborn is born at term as when the newborn is very preterm?. It is not included in the scope of the review.

Page 7. Line 30. How well represented are low- and middle-income countries in the review? Has this been measured in any way? Which countries are excluded from this search? Is there any identifiable publication bias in favour of publications with positive impact or in favour of countries with financial support from high-income countries?

VERSION 1 - AUTHOR RESPONSE

Reviewer: 1
Dr. Caitlin McGrath, Seattle Children's Hospital

Introduction:

• **First paragraph: For the sentence with "neonatal sepsis is a treatable illness", authors could also consider adding that while treatable, it can result in significant morbidity, mortality, and healthcare costs.**

Thank you for your suggestion, please see the edited sentence below.

"Although neonatal sepsis is in principle a treatable illness, and the treatment is well known, there are multiple barriers to preventing, identifying, and managing sepsis in LMIC. Due to these challenges, neonatal sepsis in LMIC frequently results in significant mortality, morbidity and healthcare costs.[2]"

• **Fourth paragraph: I would consider rewording the sentence starting with "Klebsiella" and ending with "healthcare-associated infections" to add some clarity or further detail. For example, rather than saying the "increasing problem of HAI" one could perhaps comment on the changing epidemiology of infection timing, etc.**

Thank you for your comments. Please see the edited sentence below with additional suggested detail.

"Klebsiella has traditionally been associated with late onset sepsis (sepsis >72hrs after delivery) and is associated with healthcare associated infections, however it is now increasingly being identified in the first 24 hours after birth, indicating a change in the epidemiology of the timing of onset of infection with healthcare associated infections being transmitted in the immediate hours after birth in the labour and postnatal wards.[9]"

• **NNU: Would define this at time of first use in both the abstract and introduction.**

Thank you, these have been added to line 4 of the abstract and the fifth paragraph of the introduction.

Charting section:

• **Would spelling out/defining the acronyms used in this section.**

Thank you, in the charting section under ‘Data for studies relevant to Q1’ we have edited the text to spell out in full:

“What form did parental involvement take E.g. survey, focus group discussions, in depth interviews, workshops.”

Reviewer: 2

Dr. Salvador Piris-Borregas , 12 de Octubre University Hospital

Page 3. General comment about the introduction. At no point in the introduction do the authors show possible strategies to reduce the nosocomial infection rate including the involvement of families. What strategies are they referring to? How can families be trained? Is the way families are trained systematised for all families equally or are individualised strategies tailored to the family situation being sought?

Thank you for your comment. I have added results from a scoping review on infection prevention and control bundles in LMIC NNU, that highlights possible strategies that could be taken, as well as the lack of evidence on involving families that is currently available. It is anticipated that the results of this review will highlight further possible strategies. We have edited the introduction paragraph as below.

“It is increasingly urgent to improve IPC in LMIC neonatal units (NNU) and reduce transmission of infections. One pathway to improvement that has yet been underexplored is the collaboration with families to build an infection prevention and control (IPC) intervention. Families are constantly present on neonatal units, and much of the hands-on care for their newborns is given by them. This provision of care, however, is informal with limited education for families in how to safely provide care for their newborns.[4,12] Care practices that require hygiene measures (e.g. handwashing, feeding, changing diapers) are often informally adopted through peer learning among mothers of newborns.

A scoping review on bundles of care addressing HCAI in LMIC NNUs both highlights some strategies that may be used to involve families in IPC, but also the very limited involvement of parents thus far. Identified IPC interventions were analysed by each part of an intervention, called an ‘element’. In total four separate elements were found from 44 papers that related to parental involvement. Two elements related to feeding, one to skin-to-skin contact and one to empowering mothers to engage in routine care.[13]”

Page 3. Line 27. Please, clarify the reason why the Access to the blood culture is inconsistent. It is only a matter of resources? Could be also a matter of infection protocols routines implemented in the neonatal units?

Blood culture access is inconsistent principally due to a lack of resources, but with other contributing factors. In a recent study reviewing antibiotic use and blood culture access across Kenya, Malawi, Nigeria and Tanzania, 23/61 hospitals did not have the

availability of blood cultures. However, even where blood cultures were available, coverage was often low (<50%). This was suggested to be due to challenges in the supply of consumables required for blood cultures, or lack of staff with the specialised skill, or relative cost of processing a blood culture within the healthcare systems. These challenges create inconsistent access and prevents them from becoming embedded in routine care for newborns in these settings.

Reference: Murless-Collins, S., Kawaza, K., Salim, N. *et al.* Blood culture versus antibiotic use for neonatal inpatients in 61 hospitals implementing with the NEST360 Alliance in Kenya, Malawi, Nigeria, and Tanzania: a cross-sectional study. *BMC Pediatr* **23** (Suppl 2), 568 (2023). https://doi.org/10.1186/s12887-023-04343-0

We have edited the text as follows: *"Blood cultures are recognised as the current gold standard required to confirm the diagnosis of sepsis and identify the causative pathogen – in LMIC blood culture access is limited and inconsistent. This is in part due to a lack of resources, but also due to challenges with the supply chain of consumables, availability of staff with the specialist skills necessary and relative cost of processing a blood culture within the healthcare system[5,6]. Where blood cultures are available there is often a significant delay in results or high rates of contamination, both impacting the clinical use and challenging interpretation."*

Page 3. Line 39. Please make reference in the introduction to the causes that may explain why 69% of cultures isolate gram-negative microorganisms that are not treated with the antibiotics recommended by the WHO.

Thank you, we have added an additional sentence to summarise a previous a systematic review on AMR in sub-Saharan Africa to the text to clarify:

"A meta-analysis reviewing the aetiology of invasive bacterial infections in neonates in sub-Saharan Africa indicated that in this region, Gram negative bacteria are the predominant cause of early onset sepsis, and combined with increasing antimicrobial resistance, this leads to a limited effectiveness of WHO regimens. This has, in part, been driven by antimicrobial use, for example, in Malawi rates of resistance of Gram-negative pathogens to all first-line antimicrobials has increased dramatically since the introduction of ceftriaxone in the early 2000s"

References:

Okomo U, Akpalu ENK, Le Doare K, Roca A, Cousens S, Jarde A, Sharland M, Kampmann B, Lawn JE. Aetiology of invasive bacterial infection and antimicrobial resistance in neonates in sub-Saharan Africa: a systematic review and meta-analysis in line with the STROBE-NI reporting guidelines. Lancet Infect Dis. 2019 Nov;19(11):1219-1234. doi: 10.1016/S1473-3099(19)30414-1.

Pui-Ying Iroh Tam, Patrick Musicha, Kondwani Kawaza, Jenifer Cornick, Brigitte Denis, Bridget Freyne, Dean Everett, Queen Dube, Neil French, Nicholas Feasey, Robert Heyderman, Emerging Resistance to Empiric Antimicrobial Regimens for Pediatric Bloodstream Infections in Malawi

(1998–2017), *Clinical Infectious Diseases*, Volume 69, Issue 1, 1 July 2019, Pages 61–68, https://doi.org/10.1093/cid/ciy834

Page 4. Line 43.Participants. Do the authors think that the measures to train families should be the same when the newborn is born at term as when the newborn is very preterm?. It is not included in the scope of the review.

There are likely to be different approaches required for preterm and term newborns. However, there will also be measures that are appropriate for all i.e. handwashing, visiting times, feeding and changing. This review will include both interventions for preterm and term newborns.

Edited sentence:
"The target population of this review are guardians (parents, families, or non-medical caregivers) of both term and preterm neonates in hospital in low- and middle-income countries."

Page 7. Line 30. How well represented are low- and middle-income countries in the review? Has this been measured in any way? Which countries are excluded from this search? Is there any identifiable publication bias in favour of publications with positive impact or in favour of countries with financial support from high-income countries?

The Medline filter for low- and middle-income countries has been used in this review. This filter was built based on the 2021 World Bank Lending groups, and on review of the lending groups over the last four years there are no LMIC countries from the current year (2024) that are excluded from the search due to changes in grouping. Several countries were included that have moved from upper-middle in 2021 to high-income in 2024 (American Samoa, Bulgaria, Guyana and Russia), but no studies were identified from these countries.

Within the analysis the authors plan on reviewing which countries most publications are from, and will consider funding sources within this to identify if there is a clear pattern of investment, or if there was particular investment in certain countries.
